# Genome-Wide Analyses of Heat Shock Protein Superfamily Provide New Insights on Adaptation to Sulfide-Rich Environments in *Urechis unicinctus* (Annelida, Echiura)

**DOI:** 10.3390/ijms23052715

**Published:** 2022-02-28

**Authors:** Danwen Liu, Zhenkui Qin, Maokai Wei, Dexu Kong, Qiaojun Zheng, Shumiao Bai, Siyu Lin, Zhifeng Zhang, Yubin Ma

**Affiliations:** 1 Ministry of Education Key Laboratory of Marine Genetics and Breeding, College of Marine Life Sciences, Ocean University of China, Qingdao 266003, China; liudanwen@stu.ouc.edu.cn (D.L.); qinzk@ouc.edu.cn (Z.Q.); weimaokai@stu.ouc.edu.cn (M.W.); 11170611024@stu.ouc.edu.cn (D.K.); zqj@stu.ouc.edu.cn (Q.Z.); 21190611038@stu.ouc.edu.cn (S.B.); 21190631148@stu.ouc.edu.cn (S.L.); 2 Key Laboratory of Tropical Aquatic Germplasm of Hainan Province, Sanya Oceanographic Institution, Ocean University of China, Sanya 572000, China

**Keywords:** heat shock protein, environmental adaptation, sulfide, genome, *Urechis unicinctus*

## Abstract

The intertidal zone is a transitional area of the land-sea continuum, in which physical and chemical properties vary during the tidal cycle and highly toxic sulfides are rich in sediments due to the dynamic regimes. As a typical species thriving in this habitat, *Urechis unicinctus* presents strong sulfide tolerance and is expected to be a model species for sulfide stress research. Heat shock proteins (HSPs) consist of a large group of highly conserved molecular chaperones, which play important roles in stress responses. In this study, we systematically analyzed the composition and expression of HSPs in *U. unicinctus*. A total of eighty-six HSP genes from seven families were identified, in which two families, including sHSP and HSP70, showed moderate expansion, and this variation may be related to the benthic habitat of the intertidal zone. Furthermore, expression analysis revealed that almost all the HSP genes in *U. unicinctus* were significantly induced under sulfide stress, suggesting that they may be involved in sulfide stress response. Weighted gene co-expression network analysis (WGCNA) showed that 12 HSPs, including 5 sHSP and 4 HSP70 family genes, were highly correlated with the sulfide stress response which was distributed in steelblue and green modules. Our data indicate that HSPs, especially sHSP and HSP70 families, may play significant roles in response to sulfide stress in *U. unicinctus*. This systematic analysis provides valuable information for further understanding of the function of the HSP gene family for sulfide adaptation in *U. unicinctus* and contributes a better understanding of the species adaptation strategies of marine benthos in the intertidal zone.

## 1. Introduction

Heat shock proteins (HSPs) are a set of evolutionarily conserved molecular chaperones that exist widely, from bacteria to animals. HSPs were first identified in the fruit fly (*Drosophila melanogaster*) exposed to a severely heat-shocked environment [1,2,3,4]. Subsequent research showed that a wide range of environmental stressors, such as hypoxia, salinity, heavy metals, and toxic compounds can also trigger intracellular HSP production at a high level [5,6,7]. HSP functions are particularly important for cell survival under stress, and are involved in maintaining protein functional conformation, facilitating protein transport through membrane channels, and preventing the aggregation of non-native proteins [8]. Generally, HSPs are divided into seven different families based on sequence homology and their molecular weight, including HSP110, HSP90, HSP70 (HSPA), HSP60, HSP40 (DNAJ), HSP10, and sHSP (HSPB) [9]. The similarity of amino acid sequences among the different HSP families is relatively low, whereas the sequences among the members in the same family are highly correlated and share evolutionarily conserved motifs [5,10]. HSPs in eukaryotes exhibit a variety of family characteristics that differ from each other in the number of members, gene structure, sub-cellular location, and expression level [11]. For example, the number of members in the HSP70 family varies from 15 in human (*Homo sapiens*) to 88 in oyster (*Crassostrea gigas*) [12,13]. The wide variation in HSP composition and characteristics may reflect environmental adaption during the evolution of species.

The intertidal zone is a peculiar and dynamic coastal environment between land and ocean, and is one of the most productive coastal ecosystems with estuaries [14]. As an ecotone, it represents a particular environment characterized by changing environmental factors like temperature, oxygen concentration, ultraviolet radiation, etc. [15] The intertidal zone harbors rich and diverse species and these organisms may have significant adaptability to environments and possess advanced molecular mechanisms against varied stressors. Notably, intertidal sediments generally act as important sinks for pollutants in coastal environments [16,17]. Recently, growing evidence has confirmed the importance of HSPs in response to various environmental pollutants and stressors [18,19,20,21,22,23,24]. Sulfide, a sum of H_2_S, HS^−^, and S^2−^, is widely distributed in intertidal sediments. The hydrogen sulfide concentration especially can accumulate up to 65 μM during low tides in the intertidal sediments [25]. Excessive H_2_S is toxic to organisms, due to effects such as inhibiting the cytochrome *c* oxidase of the mitochondrial respiratory chain and modifying oxygen transport proteins, which reduces their oxygen affinity [26,27]. At present, only a few HSPs have been demonstrated to respond to sulfide stress in several marine invertebrates based on the results of RT-qPCR and RNA-seq, including *hsp70* from marine crabs (*Charybdis japonica*) [18], the HSP70 family gene (*hspa1/8*, *hspa5*, *hspa12a*) and the HSP40 family gene (*dnaja1*, *dnajb1*) in ark shells (*Anadara broughtonii*) [21], as well as *hsp21*, *hsp70*, and *hsp90* from white shrimp (*Litopenaeus vannamei*) [20]. Therefore, systematic analysis of the HSP gene family in response to sulfide stress is essential to illuminating the adaptation strategies of marine benthos in the intertidal zone.

The Echiura worm *Urechis unicinctus* is a benthic organism inhabiting U-shaped burrows in the intertidal zone in China, Korea, and Japan, and is a delicious seafood with high nutritional value [28]. Previous researchers have revealed that *U. unicinctus* can metabolize and utilize environment sulfide, and exhibits a strong sulfide tolerance as well as surviving well in a sulfide-rich environment [29,30,31,32,33,34,35]. To better understand the composition characteristics of the HSP superfamily and the mechanism of HSP function in the sulfide tolerance of burrowing animals, we identified members of the HSP superfamily according to our whole genome data of *U. unicinctus*, and analyzed the evolutionary relationship of HSPs by comparing the genome data with closely related species. Furthermore, the expression profiles of the HSP genes were analyzed based on transcriptome data in *U. unicinctus* after sulfide exposure. Our data will be valuable for illuminating the mechanism of *U. unicinctus* sulfide tolerance, and also give us a better understanding of the evolution of the HSP gene superfamily in intertidal animals.

## 2. Results and Discussion

### 2.1. Identification and Characterization of the HSPs in U. unicinctus

In this study, all the putative HSP sequences were obtained by an HMMER search, and then confirmed by the presence of characteristic conserved domains using Pfam, SMART, and NCBI-CDD. Annotation and nomenclature of these HSP genes were completed based on amino acid sequence similarities and phylogenetic analysis (Supplementary Materials Figure S1 and Table S1). A total of 86 putative HSP genes were identified in the *U. unicinctus* genome, which included 1 HSP10, 27 sHSP, 30 HSP40, 1 HSP60, 23 HSP70, 3 HSP90, and 1 HSP110 (Table S1). Their biochemical properties (e.g., length, molecular weight, and isoelectric point) are presented in Table S1.

**Figure 1:**
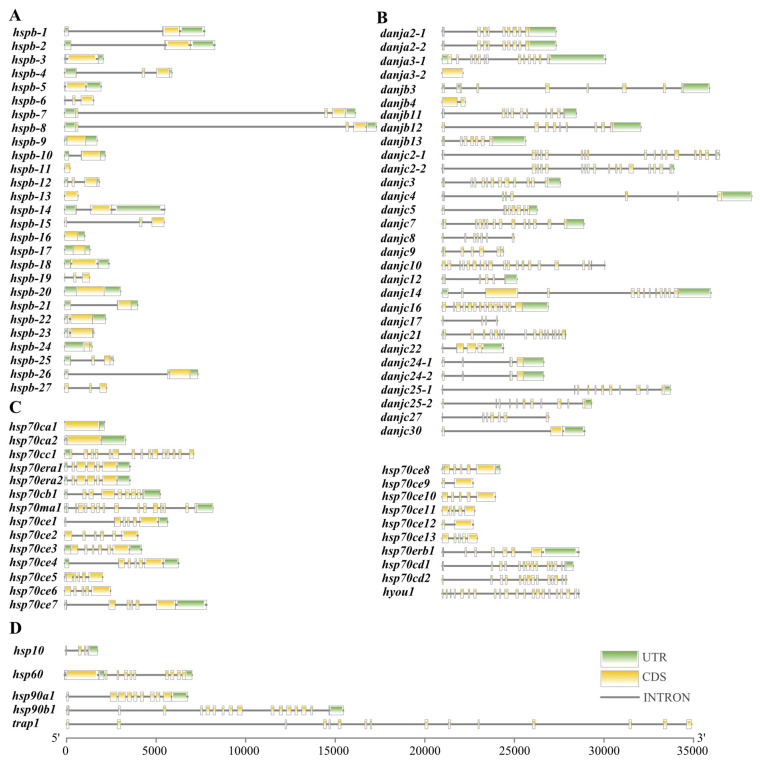
Gene structure of the HSPs in *U. unicinctus*. (**A**) sHSP; (**B**) HSP40; (**C**) HSP70/110; (**D**) HSP10, HSP60, and HSP90.

In general, most of the genes within the same family shared similar gene structures in terms of either exon length or intron number [36]. In *U. unicinctus*, similar characteristics were also presented, such as in HSP110 genes with 24 introns, and most HSP40 and HSP90 genes with more than 5 introns. The number of introns for sHSP and HSP70 genes was generally few in *U. unicinctus* and some of them were even intron less, for example, most of the sHSPs had one to two introns, and ten of them were without introns (Figure 1). Intron-less genes are generated mostly by retro-transposition of mRNA and may be advantageous to accelerating the transcription process and rapidly moving from the nucleus to the cytoplasm without splicing as an acute response occurs [37]. It has been found previously that genes rapidly activated in stress responses tend to evolve a decreased intron density [38]. This means that *U. unicinctus* probably prefers rapid transcription of sHSP and HSP70 genes to respond to environmental changes.

**Figure 2:**
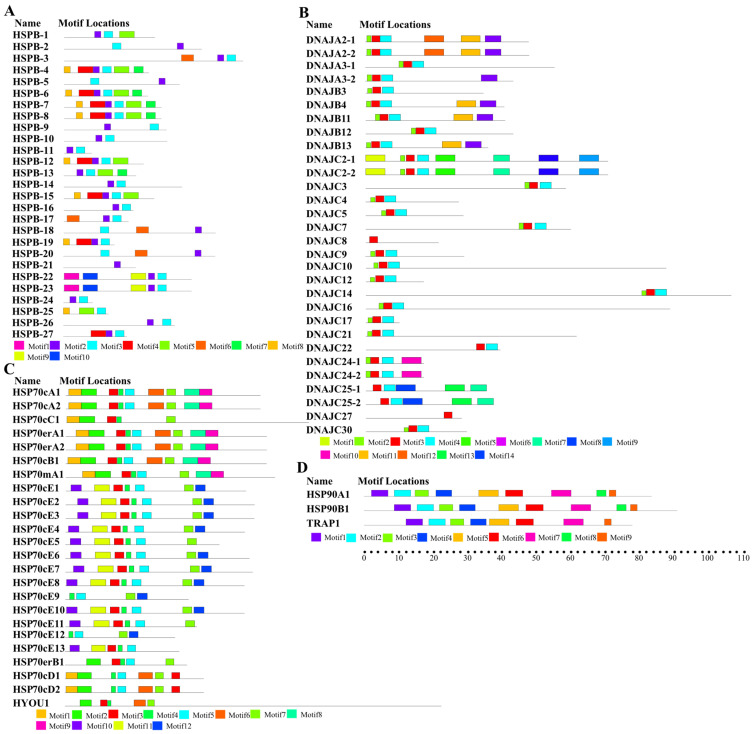
Motif composition of the HSP members in *U. unicinctus*. (**A**) sHSP; (**B**) HSP40; (**C**) HSP70/110; (**D**) HSP90. All motifs were identified by MEME 5.1.1 using the complete amino acid sequences. Different motifs are indicated by the different colors. The detailed sequence information of different motifs is shown in Table S3.

To assess the diversity and similarity of motif compositions among the different HSP genes, a conserved motif analysis was performed by MEME. The results showed that some motifs corresponded to components of the known HSP domains based on the analysis of Pfam, SMART, and NCBI-CDD. For instance, the motifs 2 and 3 in Figure 2A together formed a highly conserved alpha-crystallin domain in the sHSP family, which is the structural basis for the biological function of sHSPs. Besides that, the motifs 3 and 4 in Figure 2B were annotated as the DnaJ domain in the HSP40 family. Similarly, the signature motifs of HSP70 were identified in motifs 1, 5, and 7 (Figure 2C). Furthermore, the motifs 2 and 3 in Figure 2D corresponding to the ATPase domain at the N-terminus were found in all HSP90 proteins from *U. unicinctus*. The similar motif compositions provided structural similarity for HSP proteins, and might further lead to functional similarity. In *U. unicinctus*, HSP90 family members demonstrated the same motif composition, reflecting their high evolutionary conservation, whereas other HSP families presented variable motif patterns. In the HSP70cE subfamily particularly, approximately half (13 of 23) of HSP70 family genes exhibited quite different motifs. Motifs 3, 4, 5, and 7 were presented in almost all HSP70 proteins, whereas the motifs 10, 11, and 12 existed only in the HSP70cE subfamily (Figure 2C). The difference between HSP70cE subfamily members and other HSP70 genes was consistent with the findings of phylogenetic analysis where the HSP70cE subfamily was distantly related to other HSP70s (Figure S1). The conserved motif analysis results indicated that the HSP70cE subfamily was the least-conserved subfamily of the HSP70 family in *U. unicinctus*, suggesting that the HSP70cE subfamily may have unique biological functions.

### 2.2. Genomic Location and Gene Duplication Events in the HSP Gene Superfamily

To further investigate the genomic distribution and gene duplication of the identified HSPs, these genes were plotted on chromosomes based on the genomic database. In *U. unicinctus*, all the HSP genes were unevenly distributed on chromosomes besides *danjc25* which was anchored on un-assembled scaffold272. The largest number of HSP genes was located on chromosome 2 which contained 16 HSP genes (Figure S2), 12 of which belonged to the sHSP gene family. Tandem duplicated genes are defined by the criteria that are located within a 100-kb distance and separated by five or less genes [39]. According to the criteria, we found duplicated HSPs were mostly ascribed to tandem duplication, including twelve sHSPs (44%), ten HSP70s (37%), and eight HSP40s (27%). Gene duplication may always be significant to the evolution and functional diversification of gene families [40]. The duplication and divergence of HSP genes might help animals adapt to varied stress conditions [41]. Therefore, we suggest that the gene duplication of HSPs may play an important role against stress conditions and adapting to the environment in *U. unicinctus*.

### 2.3. Expansion of sHSP and HSP70 Family Genes May Be a General Biosignature for Zoobenthos

**Figure 3:**
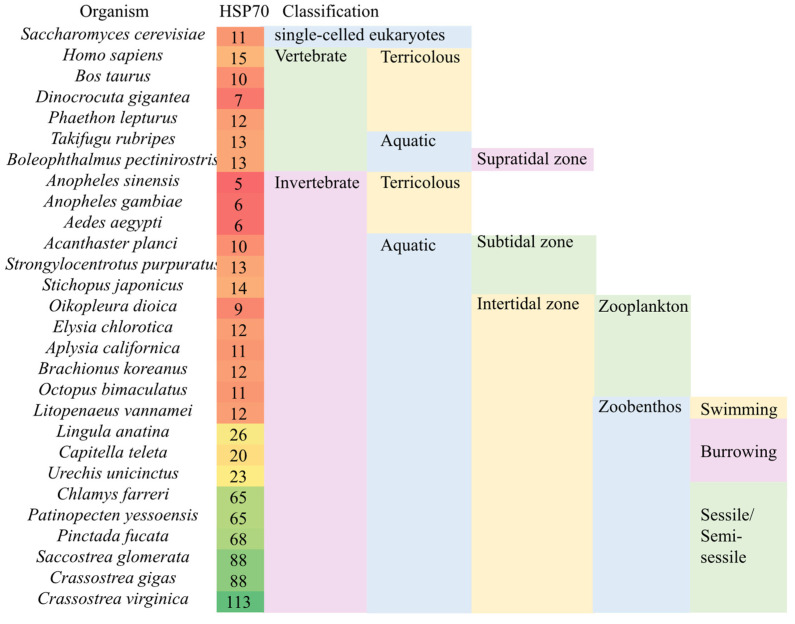
HSP70 gene copy numbers among the species in the different habitats. The number in the left column is the HSP70 gene copy number.

Gene expansion is a rapid mechanism generating additional sequences for natural selection to confer greater organismal fitness. If additional copies of the gene are beneficial, this process may be repeated to produce an expanded gene family containing many copies of related sequences [42]. In *U. unicinctus*, obvious gene expansions occurred in the sHSP and HSP70 gene families (Figure 3 and Figure S3, and Table S4).

Expansion of the sHSP family genes was significant in *U. unicinctus*; the number of gene copies was 27 and about two times more than the average of other representative species (Figure S3). Further analysis indicated that sHSP genes were also expanded three-fold more than the average of other species in *Litopenaeus vannamei* and two-fold more in *Capitella teleta* (Figure S3). *U. unicinctus*, *L. vannamei*, and *C. teleta* are three typical pollutant-tolerant benthos that live in the intertidal zone. Among them, *L. vannamei* was reported to be resistant to a variety of pollutants, including nitrite [43], ammonia [44], sulfide [21], etc. *C. teleta* is a sediment-dwelling marine polychaete that is described as a pollution indicator species and is often found in disturbed or stressed organically and sulfide-enriched environments [45,46]. Due to their expansion in three representative pollutant tolerant benthos, we suggest that sHSP genes may play pivotal roles in the adaptation to pollutants.

The HSP70 gene number in *U. unicinctus* was also expanded moderately with a copy number of 23, and about 1.5 times more than the average of other representative species (Figure 3 and Table S4). In fact, the significant expansion of HSP70 has also been reported in some intertidal sessile and semi-sessile benthic organisms, such as some bivalves, *Chlamys farreri*, *Crassostrea angulate*, and *Crassostrea virginica* [47,48,49]. In other burrowing animals, copy number analyses indicated that the HSP70 gene family was moderately expanded similarly to *U. unicinctus*, such as in *Lingula anatine* and *C. teleta* [50,51]. However, similar expansions have not been seen in free-floating and swimming intertidal animals, such as *Octopus bimaculoides*, *Elysia chlorotica*, *Aplysia californica*, and *L. vannamei* [52,53,54,55], in which HSP70 copy numbers were similar to vertebrates and widely studied model organisms, such as *Saccharomyces cerevisiae* [56] (Figure 3). The copy numbers of HSP70 family genes may be negatively correlated with the escape ability of marine invertebrates in intertidal zonation. The results implied that the expansion of HSP70 may be a characteristic of intertidal benthic invertebrates, which may improve their adaptability to the complex and changeable benthic environment during tidal cycles.

In summary, we suggest that the expansion of sHSP and HSP70 family genes in *U. unicinctus* may be an important adaptation to the benthic environment and constitute a general biosignature of environmental stress adaptation.

### 2.4. Most HSP Genes Are Involved in the Sulfide Stress Response in U. unicinctus

**Figure 4:**
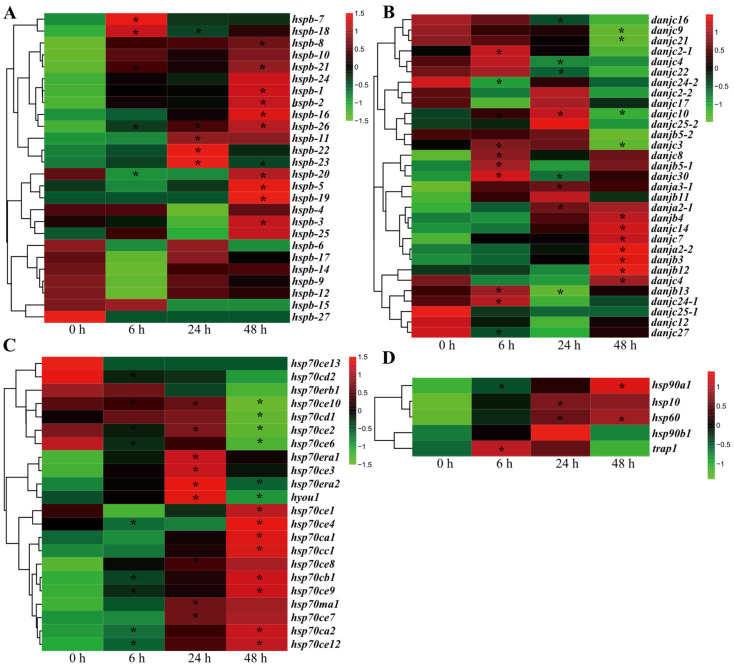
Expression of HSP superfamily genes in *U. unicinctus* exposed to sulfide based on the FPKM in the transcriptome data. (**A**) sHSP; (**B**) HSP40; (**C**) HSP70/110; (**D**) HSP10, HSP60, and HSP90. * indicates the significantly regulated HSP genes with |log2FC| > 2 and *p* < 0.05.

Sulfide is one of the most representative pollutants in the intertidal zone. To further reveal HSP function in environmental stress adaptation, we investigated the response of HSP genes under sulfide stress by in-depth transcriptomic analysis of *U. unicinctus*. The results showed that the expression levels of most HSP genes presented significant difference between the sulfide group and control (Figure 4), suggesting their potential roles in sulfide stress response. Specifically, expressions of the HSP10, HSP40, HSP60, and HSP90 genes were significantly changed after sulfide stress, although the difference trend varied across the different HSPs (Figure 4B,D); most sHSP and all the HSP70cE subfamily genes indicated upregulated expression at 48 h after sulfide stress (Figure 4A,C). Recently, HSP gene responses to sulfide stress have been reported in several species: *hsp70* in marine crabs (*Charybdis japonica*) [18]; HSP70 family genes (*hspa1/8*, *hspa5*, *hspa12a*), sHSP, and HSP70 in *Donax variabilis* [57]; and HSP40 family genes (*dnaja1*, *dnajb1*) in ark shells (*Anadara broughtonii*) [21] as well as *hsp21*, *hsp70*, and *hsp90* in white shrimp (*Litopenaeus vannamei*) [19]. In this study, we revealed for the first time on a genome-wide scale that most HSP genes in *U. unicinctus* were significantly unregulated or down-regulated after sulfide stress. Therefore, it may be a universal phenomenon that HSPs are involved in the sulfide stress of marine invertebrates.

### 2.5. sHSP and HSP70 Genes Are Vital Players in Sulfide Tolerance and Environmental Adaptation in U. unicinctus

**Figure 5:**
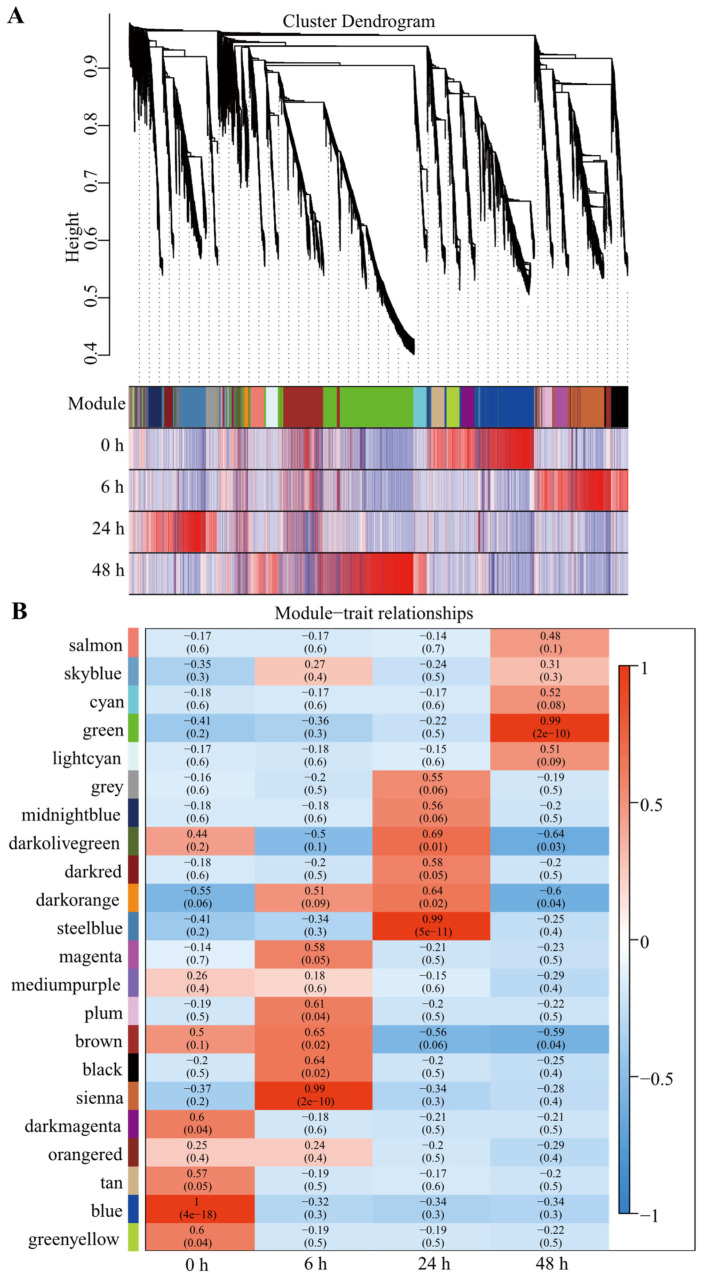
Gene co-expression analysis based on the transcriptome data from the hindgut of *U. unicinctus* exposed to sulfide stress. (**A**) Hierarchical cluster tree representing the coexpression modules identified by WGCNA. Each branch in the tree represents one gene. The major tree branches constitute 22 modules, labeled with different colors in the “Module” colored band. The remaining color bands show the correlation coefficient between individual gene and sample. (**B**) Gene module–sample association revealed by gene co-expression analysis. Each row corresponds to a module and is labeled with a different color as in (**A**). Each column corresponds to a sample. The color of each cell at the row–column intersection indicates the correlation coefficient between the module and the sample. Red represents a positive correlation, and blue represents a negative correlation. Each cell represents the correlation coefficient (upper numbers) between the module and sample, and the correlation significance (lower numbers, *p*-value), respectively.

To further investigate the potential function of HSP genes in sulfide stress response, a co-expression analysis was performed using a weighted gene co-expression network analysis (WGCNA) method that enables identification of gene co-expression modules and hub genes within modules based on gene-to-gene correlations across transcriptome datasets. In this study, there were 22 distinct modules generated, and each one was represented in a different color (Figure 5). The modules contain genes sharing highly correlated expression patterns and that are often involved in the same biological function. WGCNA analysis indicated that sienna, steelblue, and green modules were significantly correlated with sulfide stress. Furthermore, we found that HSP genes were mainly enriched in steelblue (7 HSP genes) and green (13 HSP genes) modules (Figure 5; Table S5).

To predict the possible functions or biological processes of the genes in co-expressed modules, we performed GO and KEGG analyses of genes in the steelblue and green modules. The GO analysis showed that the steelblue module and green module genes were involved in biological processes such as cellular process, metabolic process, and the response to stimulus (Figure S4). The KEGG pathway analysis showed that these genes were primarily associated with material synthesis, energy metabolism, immunity, and apoptosis (Figures S4 and S5). It is worth noting that some reported signaling pathways involved in sulfide stress response, such as phosatidylinositol-3 kinase (PI3K-Akt) [58,59] and RAS/mitogen-activated protein kinase (MAPK) signaling pathways [60,61], were also significantly enriched in the steelblue and green modules, respectively (Figure S5).

**Figure 6:**
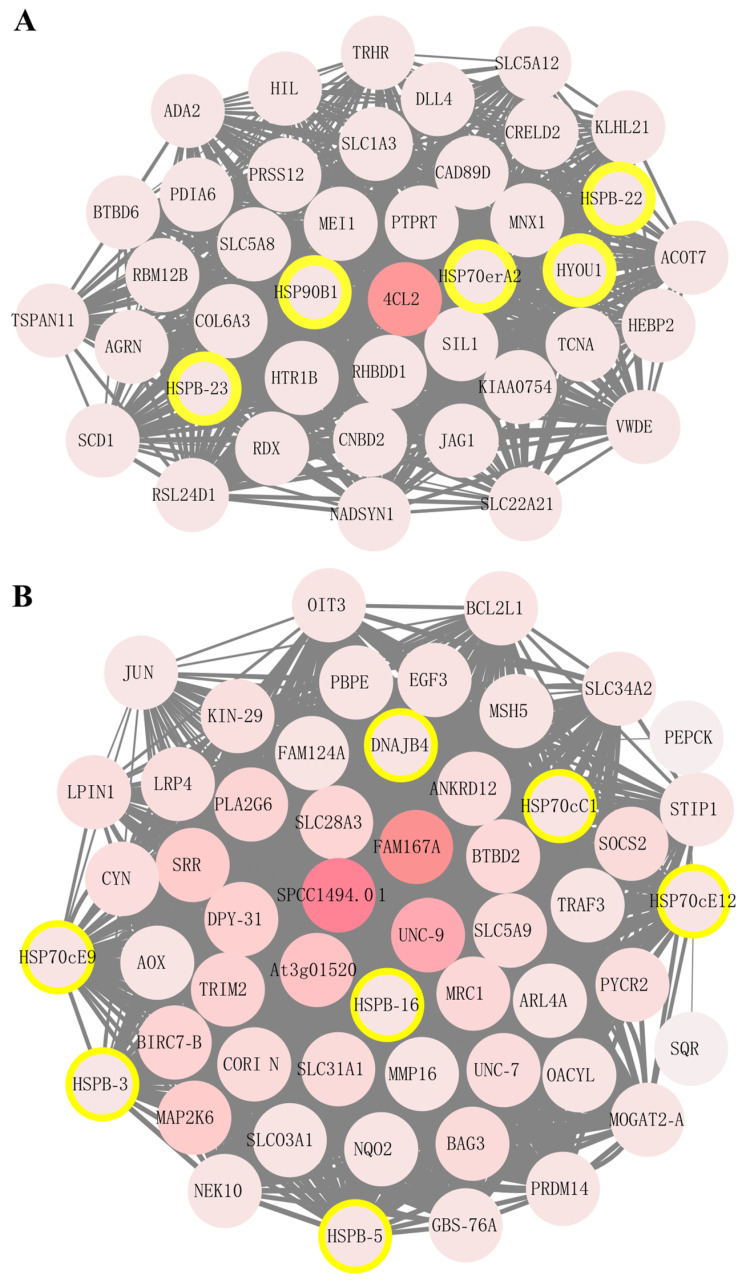
Graphical visualization of the *U. unicinctus* weighted gene co-expression network in steelblue and green modules. Each node represents a gene. The darker the red node color, the higher the degree of connectivity between the gene and the module. Hub genes were selected based on their higher connectivity, and the HSP genes were circled with a yellow border. (**A**) Steelblue module from Figure 5B; (**B**) Green module from Figure 5B. An enlarged version is shown in Figure S6.

To explore the potential interaction and function of co-expressed genes, two co-expression networks were visualized using Cytoscape software in the steelblue and green modules, respectively (Figure 6). The *4cl2* gene was identified as the hub regulation genes in the steelblue module; the genes *fam167a*, *spcc1494.01*, and *unc-9* were obtained as the hub regulation genes in the green module. It can be speculated that they may be the most important regulated genes in response to sulfide stress in *U. unicinctus* (Figure 6). These four genes have been reported as being involved in stress response and immune defense [62,63,64,65]. Here, we speculate that they may play important roles in the response to sulfide stress, and further studies are needed to clarify the potential molecular mechanisms.

Furthermore, the gene co-expression networks of WGCNA showed that five HSP genes (*hspb-22*, *hspb-23*, *hsp70era2*, *hsp90b1*, and *hyou1*) have high connectivity to the hub gene *4cl2* in the steelblue module (Figure 6A), and seven HSP genes (*hspb-3*, *hspb-5*, *hspb-16*, *hsp70cc1*, *hsp70ce9*, *hsp70ce12*, and *dnajb4*) have high connectivity to the 3 hub genes *fam167a*, *spcc1494.01*, and *unc-9* in green module (Figure 6B). Therefore, we speculated that these HSP genes may play significant roles in response to sulfide stress. Further additional approaches can be utilized in future study to validate this speculation such as proteomics [24] and Western blot, etc. Most notably, in the green module, many co-expression genes have been reported as being involved in the sulfide metabolism, including *sqr* (sulfide: quinone reductase) in sulfide oxidation metabolism, *pepck* (phosphoenolpyruvate carboxykinase) in glycolysis and gluconeogenesis, *traf3* (TNF receptor-associated factor 3) in TNF signaling pathways, *map2k6* (mitogen-activated protein kinase 6) in MAPK signaling pathways, and *xiap* (X-linked inhibtor of apoptosis protein) in NF-κB pathways [26,34,66,67]. Based on the specific co-expression network of stress-responsive genes to sulfide, we proposed a potential HSP-based gene regulatory network in response to sulfide stress in *U. unicinctus*.

Notably, in the steelblue and green modules, 12 key HSP genes mentioned above included five sHSP genes (*hspb-3*, *hspb-5*, *hspb-16*, *hspb-22*, and *hspb-23*) and four HSP70 genes (*hsp70cc1*, *hsp70era2*, *hsp70ce9*, and *hsp70ce12*), which were also the two families with gene expansion in *U. unicinctus*. Gene copy analysis among the species in the different habitats indicated the expansion of sHSP and HSP70 family genes may be an adaptation to the benthic environment and constitute a general biosignature of environmental stress adaptation. Therefore, combined with WGCNA analysis and gene expansion analysis results, we suggest sHSP and HSP70 families may play significant roles in adaptation to the complex and changeable environment in the intertidal zone.

## 3. Materials and Methods

### 3.1. Animal Materials and Treatment

Adult *U. unicinctus* with a mean length of 13.5 ± 2.1 cm were collected from the coast of Yantai (Shandong Province of China), and maintained in aerated seawater (20 °C, pH 8.0, and salinity 30 PSU) for three days. Eighteen healthy worms were randomly assigned to three sealed aquariums (six worms per aquarium) containing 30 L seawater. During the experiment, the sulfide concentration was maintained at 50 μM (equivalent to moderately polluted sediment that worms can live in without abnormalities) by adding a sulfide stock solution (10 mM Na_2_S, pH 8.0) every 2 h as necessary, and measured using the methylene blue method [68]. Three worms (one individual from each aquarium) were sampled at 0 (control), 6, 24, and 48 h after sulfide exposure, respectively. The hindgut was dissected from each worm, frozen immediately in liquid nitrogen and then stored at −80 °C for RNA extraction.

### 3.2. RNA Extraction and Illumina Sequencing

Total RNAs from the stored samples were extracted using TRIzolTM reagent (Invitrogen, Carlsbad, CA, USA) according to the manufacturer’s instructions. The RNA samples conformed to the required purity criteria and quality levels were selected for cDNA library preparation. Sequencing libraries were generated using NEBNext^®^ UltraTM RNA Library Prep Kit for Illumina^®^ (NEB, San Diego, CA, USA) following manufacturer’s recommendations, and index codes were added to attribute sequences to each sample. Non-ribosomal RNA was enriched by magnetic oligo (dT) beads and then broken into short fragments (250~300 bp) by adding fragmentation buffer. Complementary DNA (cDNA) was generated from the RNA template by a reverse transcriptase and was made double stranded by DNA polymerase. PCR products were purified using the Agencourt AMPure XP Beads (Qiagen, Hilden, Germany). Finally, the libraries were sequenced with 150 bp paired-end reads on an Illumina HiSeq X Ten platform by Novogene Company (Beijing, China).

### 3.3. Identification of HSP Members in U. unicinctus

The Hidden Markov Model (HMM) profile files of HSP10 (PF00166), HSP20 (PF00011), HSP40 (PF01556), HSP60 (PF00118), HSP70 (PF00012), and HSP90 (PF00183) were downloaded from the Pfam database. HMMER 3.0 (http://hmmer.janelia.org/, accessed on 12 May 2021) was used to search the HSPs from the *U. unicinctus* genome database (unpublished data). The default parameters were adopted and the cutoff value was set to 0.0001. All candidate genes based on the HMMER results were further examined by confirming the existence of the HSP core sequences using the NCBI-CDD (https://www.ncbi.nlm.nih.gov/cdd/ accessed on 9 June 2021) server and SMART database (http://SMART.embl-heidelberg.de accessed on 21 June 2021).

### 3.4. Multiple Alignment and Phylogenetic Analysis

The amino acid sequences of HSPs were retrieved from UniProt (http://www.uniprot.org/ accessed on 1 August 2021) and NCBI databases (http://www.ncbi.nlm.nih.gov/, accessed on 23 August 2021) (Table S2). The phylogenetic trees were constructed using the neighbor-joining method by MEGA 11.0 software (https://www.megasoftware.net/, accessed on 7 February 2022) with bootstrap values from 1000 replicates indicated at each node with the following parameters: *p*-distance and pairwise deletion. Nomenclature of all HSP genes was completed based on the amino acid sequence similarities and phylogenetic analysis, and the HSP70s were renamed according to the new nomenclature which was proposed by Yu et al. [69].

### 3.5. Sequence Analysis and Chromosomal Localization

Lengths of sequences, molecular weights, and isoelectric points of the identified HSP proteins were obtained by using tools from the ExPasy website (https://web.expasy.org/protparam/, accessed on 12 August 2021). The MEME 5.4.1 online program (https://meme-suite.org/meme/, accessed on 20 August 2021) for protein sequence analysis was used to identify the conserved motifs in the identified HSP proteins. The gene structures were obtained from the GFF annotation file of *U. unicinctus* genome and then were displayed by Gene Structure Display Server 2.0 (http://gsds.gao-lab.org/, accessed on 2 September 2021). The mapping of HSP genes’ chromosomal positions and relative distances was acquired from the *U. unicinctus* genome and displayed by TBtools [70].

### 3.6. Expression Profiles and Co-Expression Network Construction

The transcriptome raw reads were firstly filtered to obtain high-quality sequences (clean reads) by removing low quality sequences and adapter contamination. The cleaned sequences were mapped to the *U. unicinctus* genome using STAR. HTSeq was used to determine the read count of each gene, and gene expression levels were estimated as fragments per transcript kilobase per million fragments mapped (FPKM) values. Differentially expressed genes (DEGs) were defined as those with an adjusted *q*-value < 0.05 and a |log2 fold-change| > 1, as determined by DESeq. The heatmap was exhibited using “heat map” R-package.

Co-expression networks were constructed using the WGCNA package (version 1.70-3; https://cran.r-project.org/web/packages/WGCNA/index.html accessed on 20 May 2021) in R [71]. The appropriate power was determined when the index value for the reference dataset exceeded 0.9. Module-trait associations were estimated using the correlation between the module eigengene and the stress treatments. The correlation between modules and traits was analyzed by the “cor” function in the R package stats. In addition, the “corPvalueStudent” function was used to calculate the student asymptotic *p*-value for each correlation via the R package WGCNA [71,72]. The result of *p*-value < 0.05 was considered to be a significant correlation between the module and the trait. Network visualization for each module was performed using the Cytoscape software version 3.6 with a cut off of the weight parameter (obtained from the WGCNA) set at 0.55. GO term enrichment analysis in the gene modules was performed using the EnrichPipeline, and KEGG pathway enrichment analysis was performed using KOBS at *p* < 0.05 [73].

## 4. Conclusions

This is a systematic study to identify the HSP gene superfamily in *U. unicinctus* at the genomic level. A total of 86 HSP genes were identified, including one HSP110, three HSP90s, twenty-three HSP70s, one HSP60, thirty HSP40s, twenty-seven sHSPs, and one HSP10. The copy number analysis indicated that moderate expansion of HSP70 seems to be widespread in intertidal burrowing organisms, and the expansion of sHSP may be related to the pollution resistance of intertidal benthos. WGCNA analysis demonstrated that five sHSP genes and four HSP70 genes may play significant roles in sulfide adaptation. The findings of this study are useful for the further investigation of the functions of HSPs and can also contribute a better understanding of the species adaptation strategies of marine benthos in the intertidal zone.

## Data Availability

The RNA-Seq raw sequence data were deposited in the National Center for Biotechnology Information (NCBI) Sequence Read Archive (SRA) database under the accession number SRP331312.

## Supplementary Materials

The following supporting information can be downloaded at: www.mdpi.com/article/10.3390/ijms23052715/s1.
