# Supplementary figures and images for: Genome-Wide Analyses of Heat Shock Protein Superfamily Provide New Insights on Adaptation to Sulfide-Rich Environments in Urechis unicinctus (Annelida, Echiura)
Source: Int J Mol Sci. 2022 Feb 28;23(5):2715. doi: 10.3390/ijms23052715 (PMC8910992; doi:10.3390/ijms23052715)

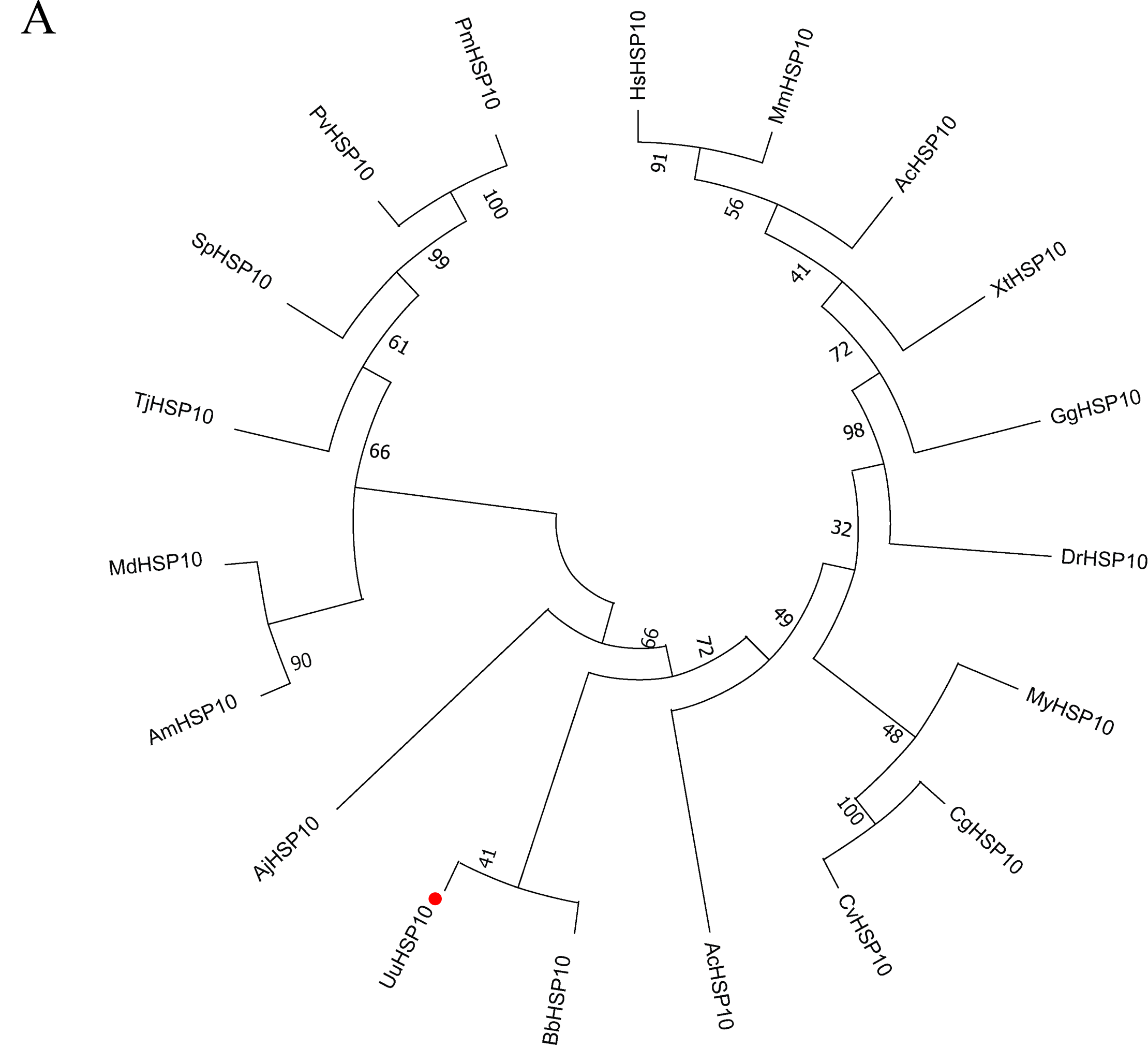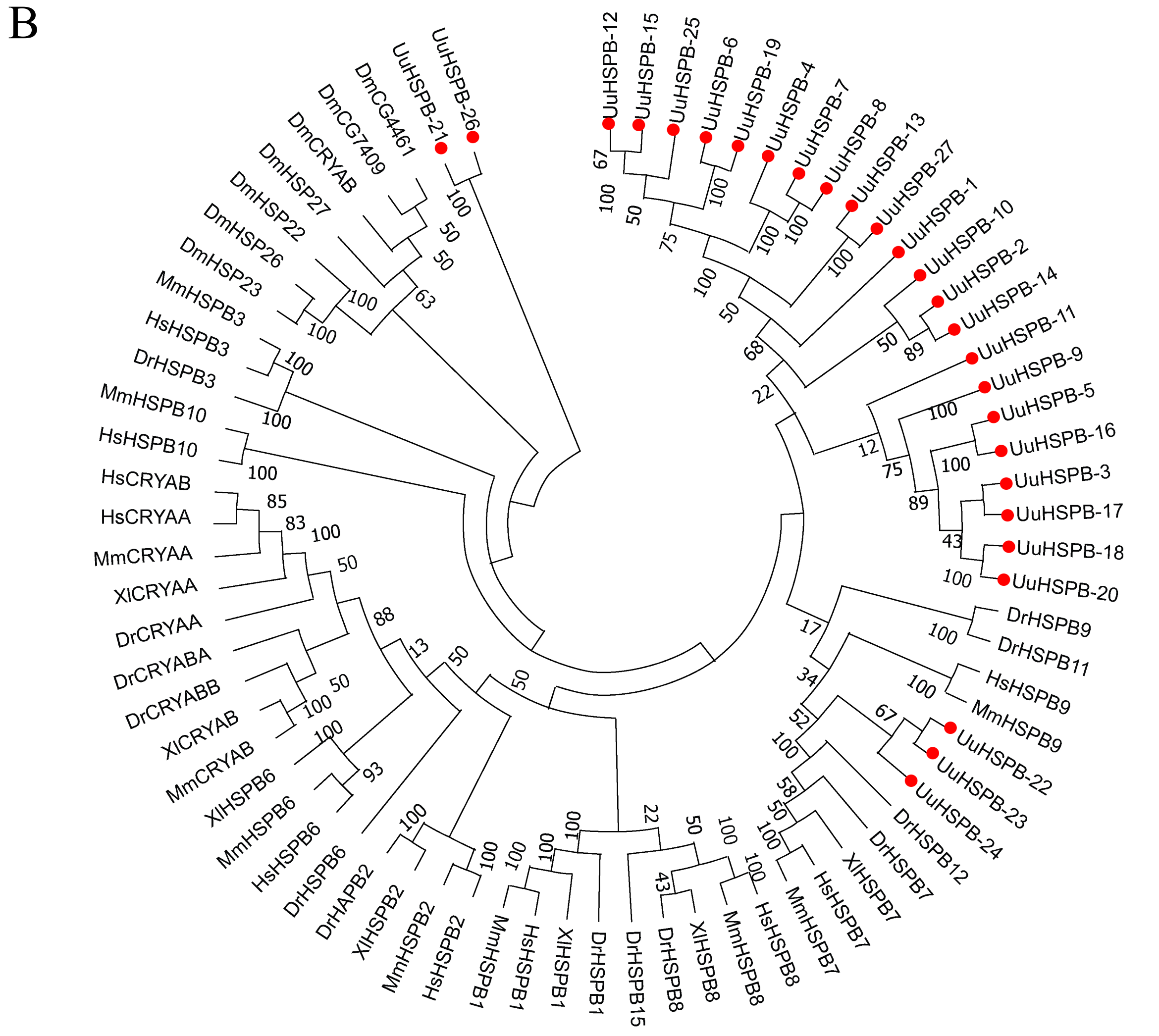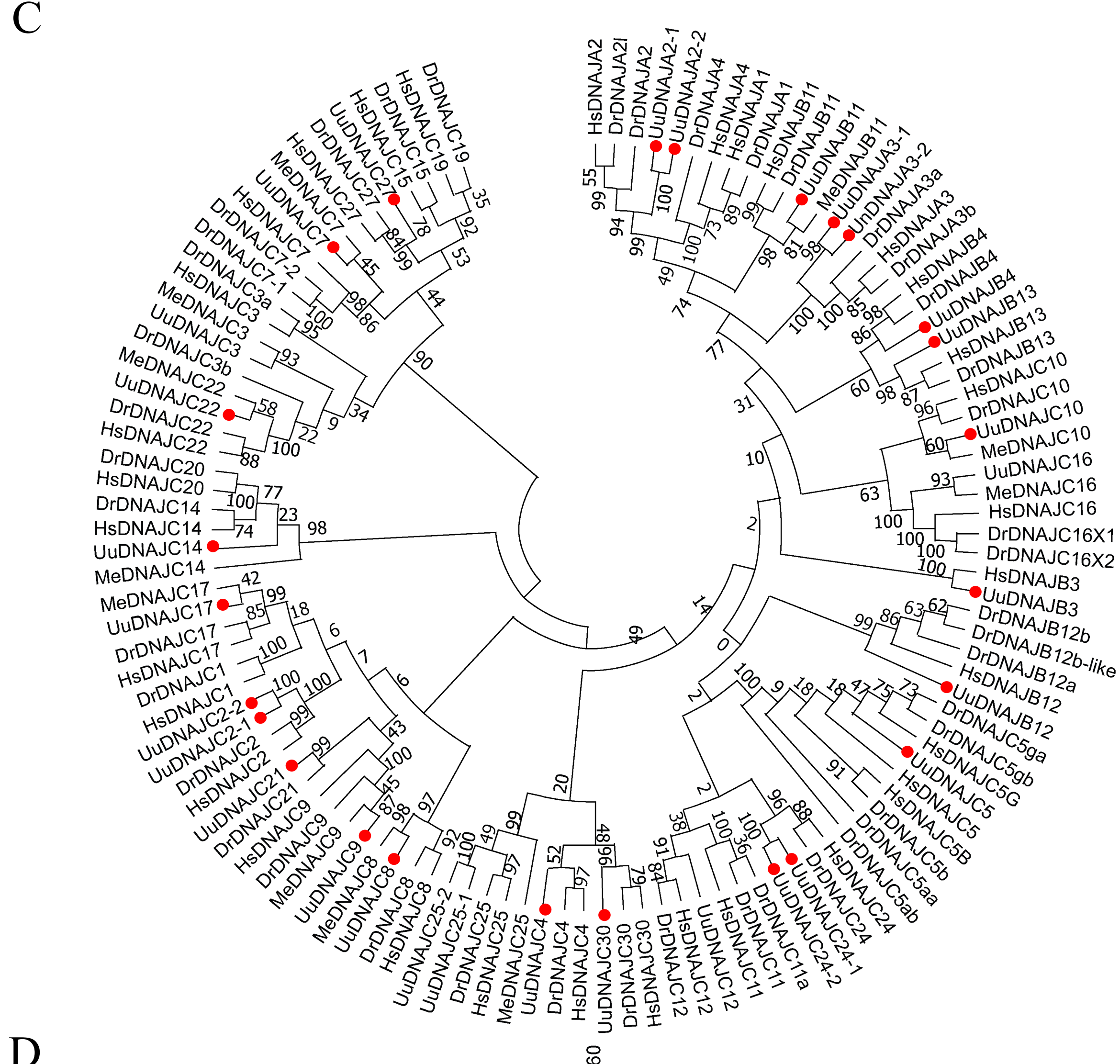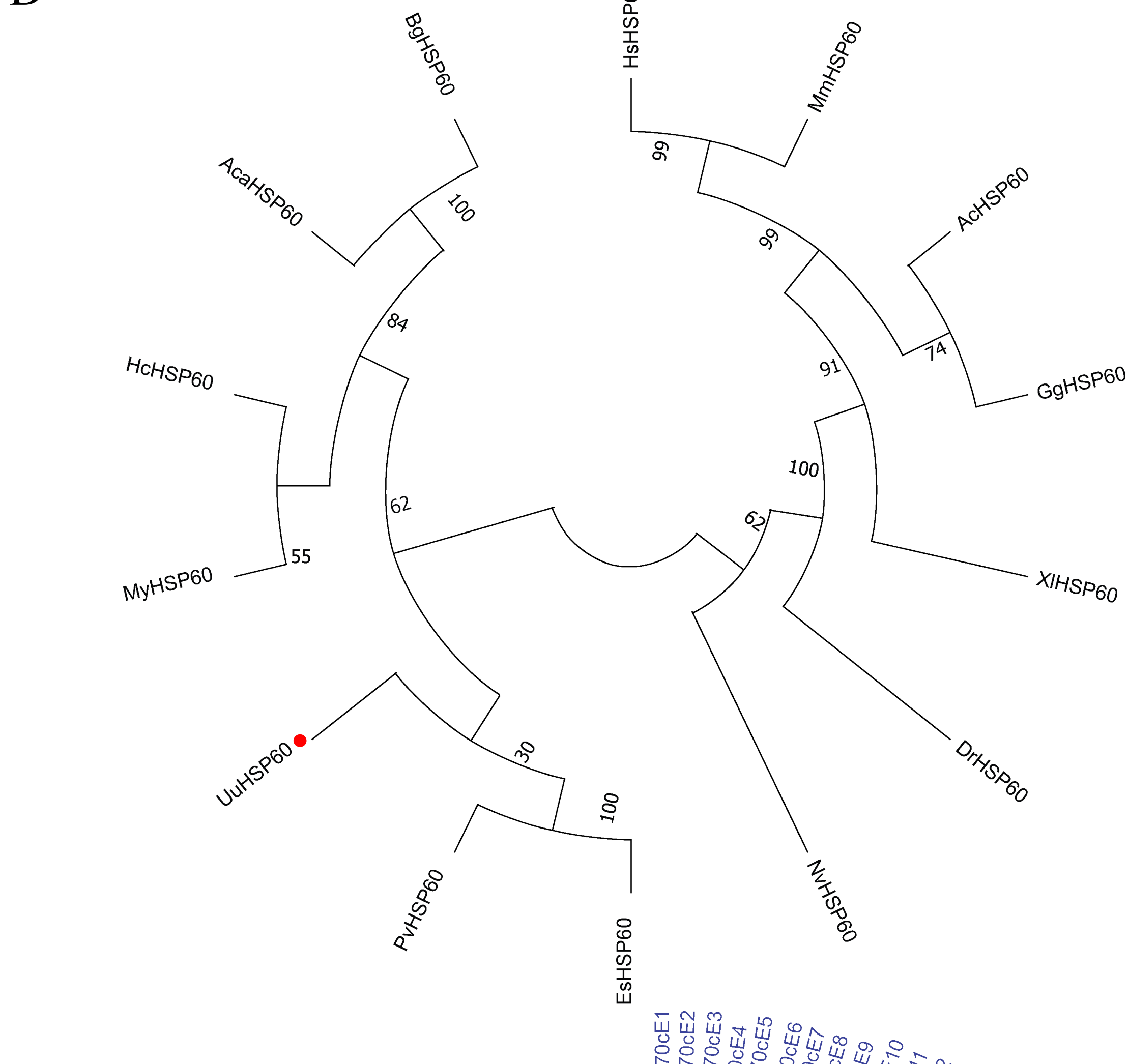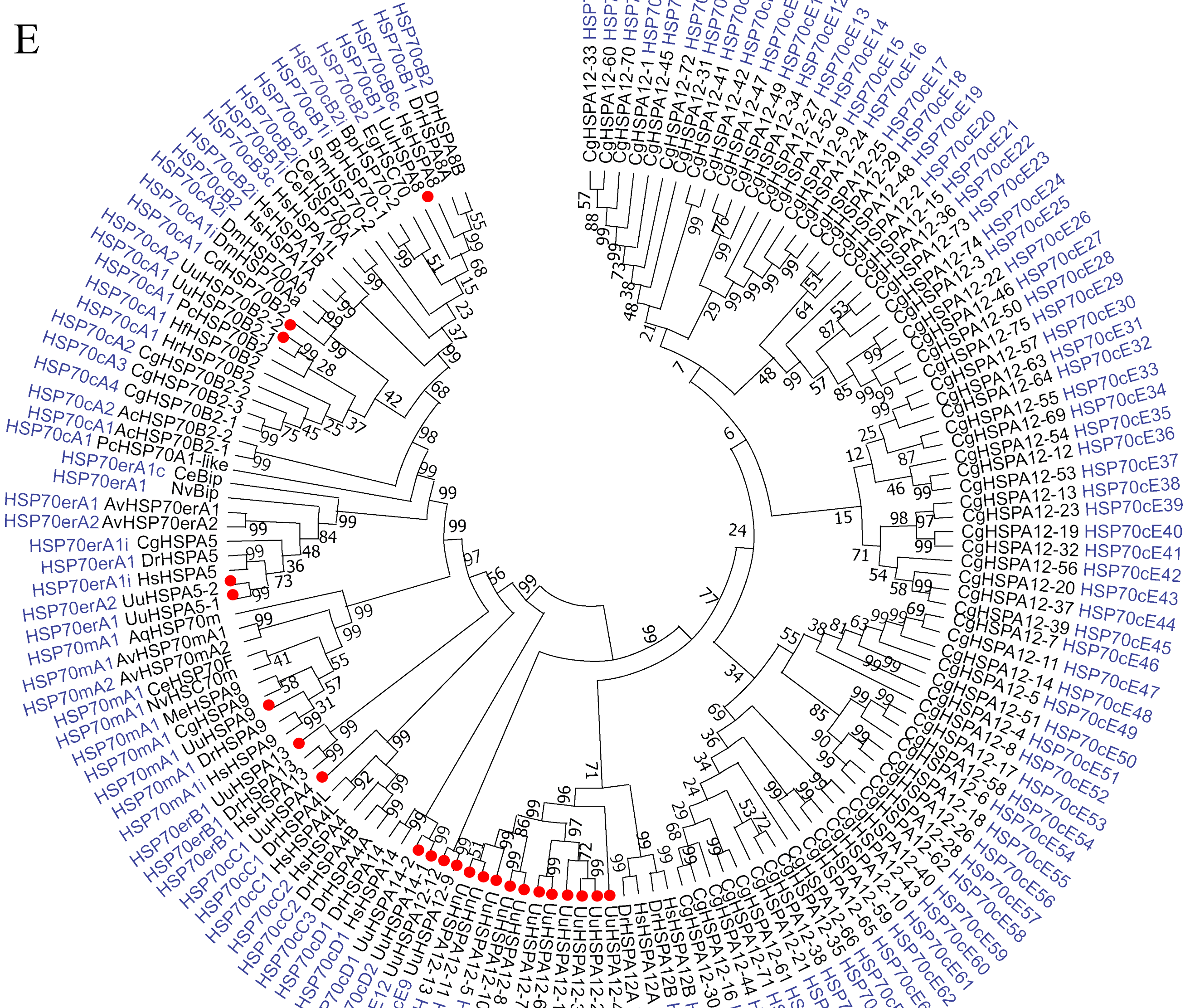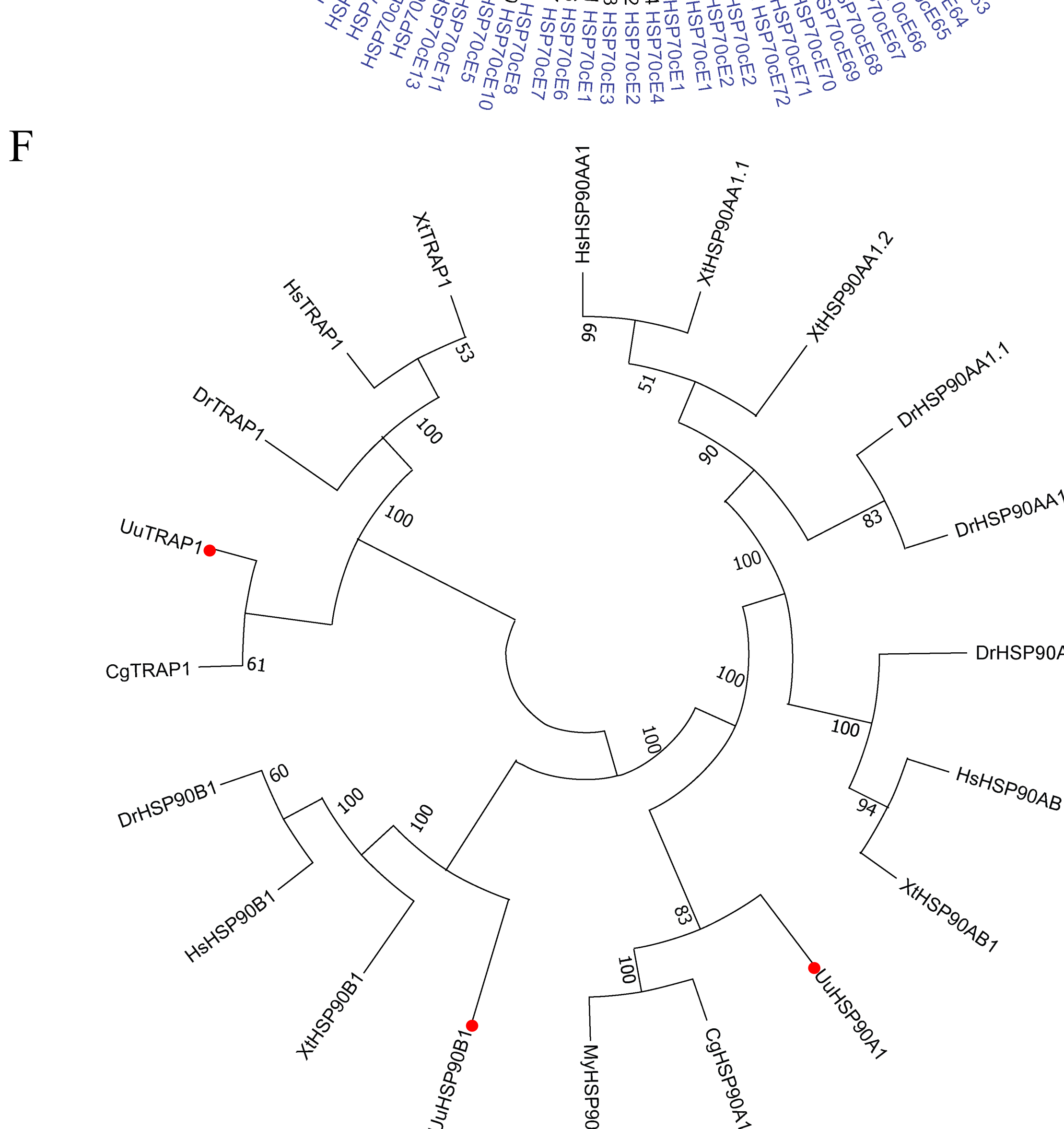

Supplement: Supplementary file 1 [file ijms-23-02715-s001.zip › Figure S1.pdf]

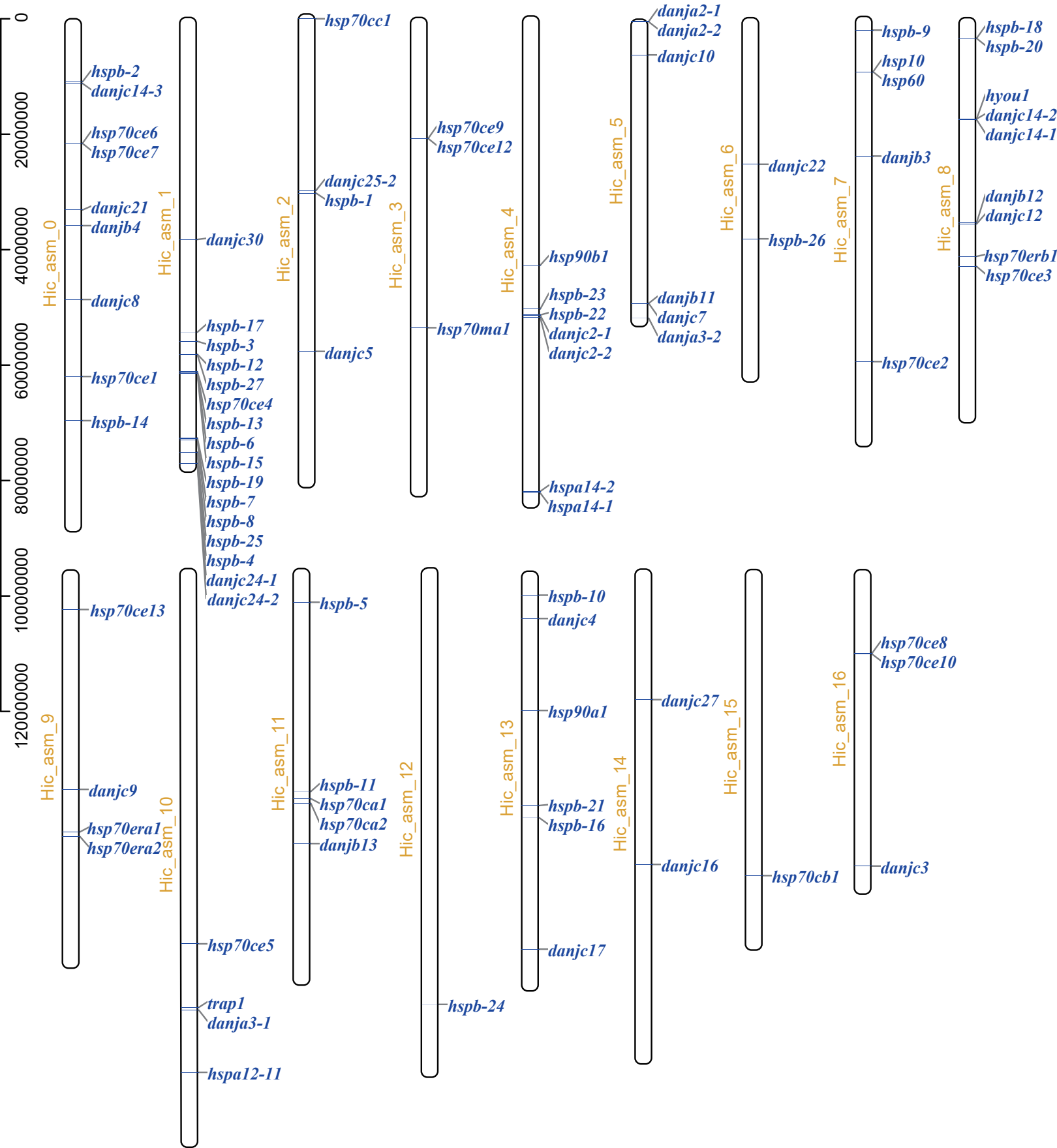

Supplement: Supplementary file 1 [file ijms-23-02715-s001.zip › Figure S2.pdf]

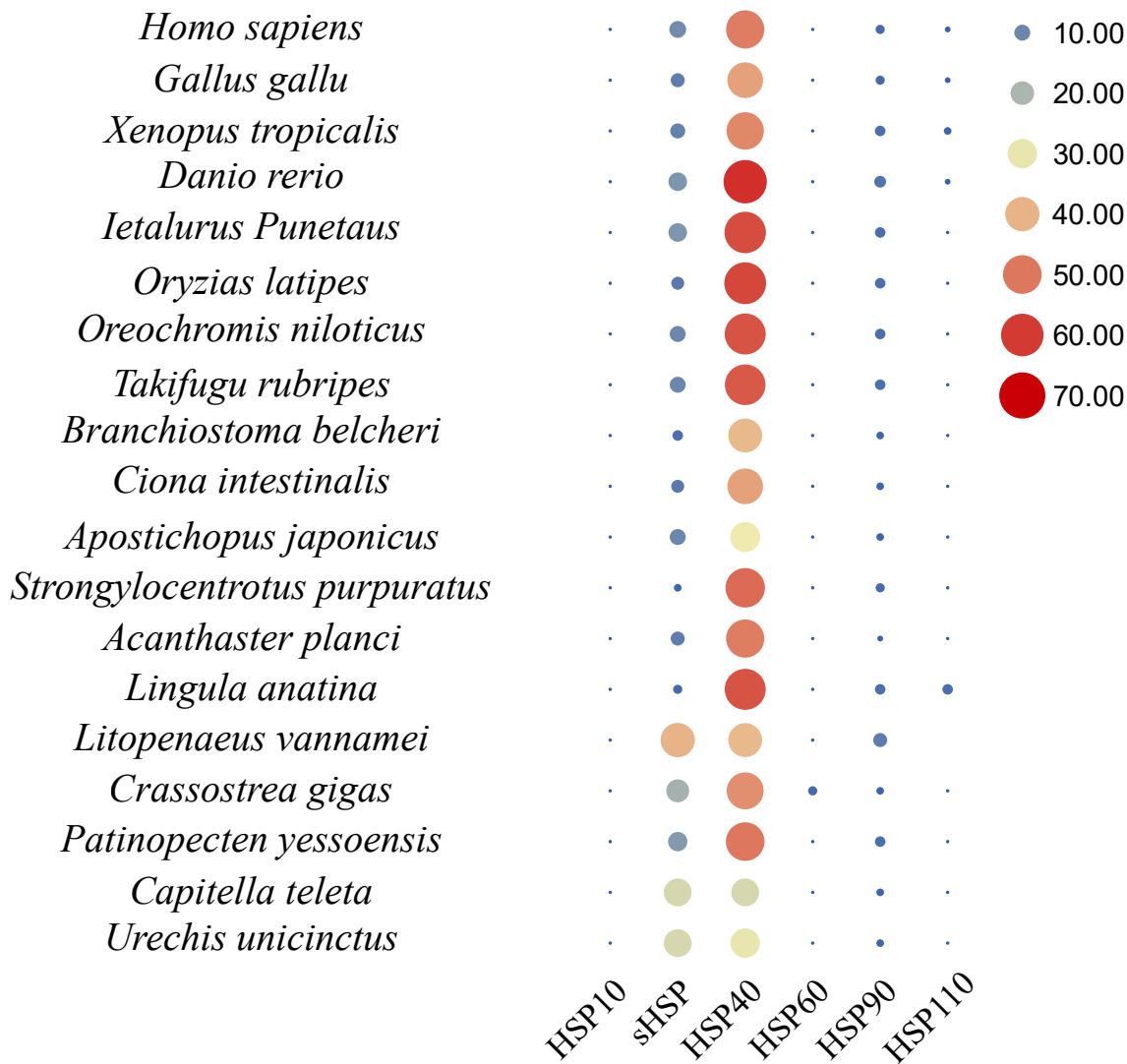

Supplement: Supplementary file 1 [file ijms-23-02715-s001.zip › Figure S3.pdf]

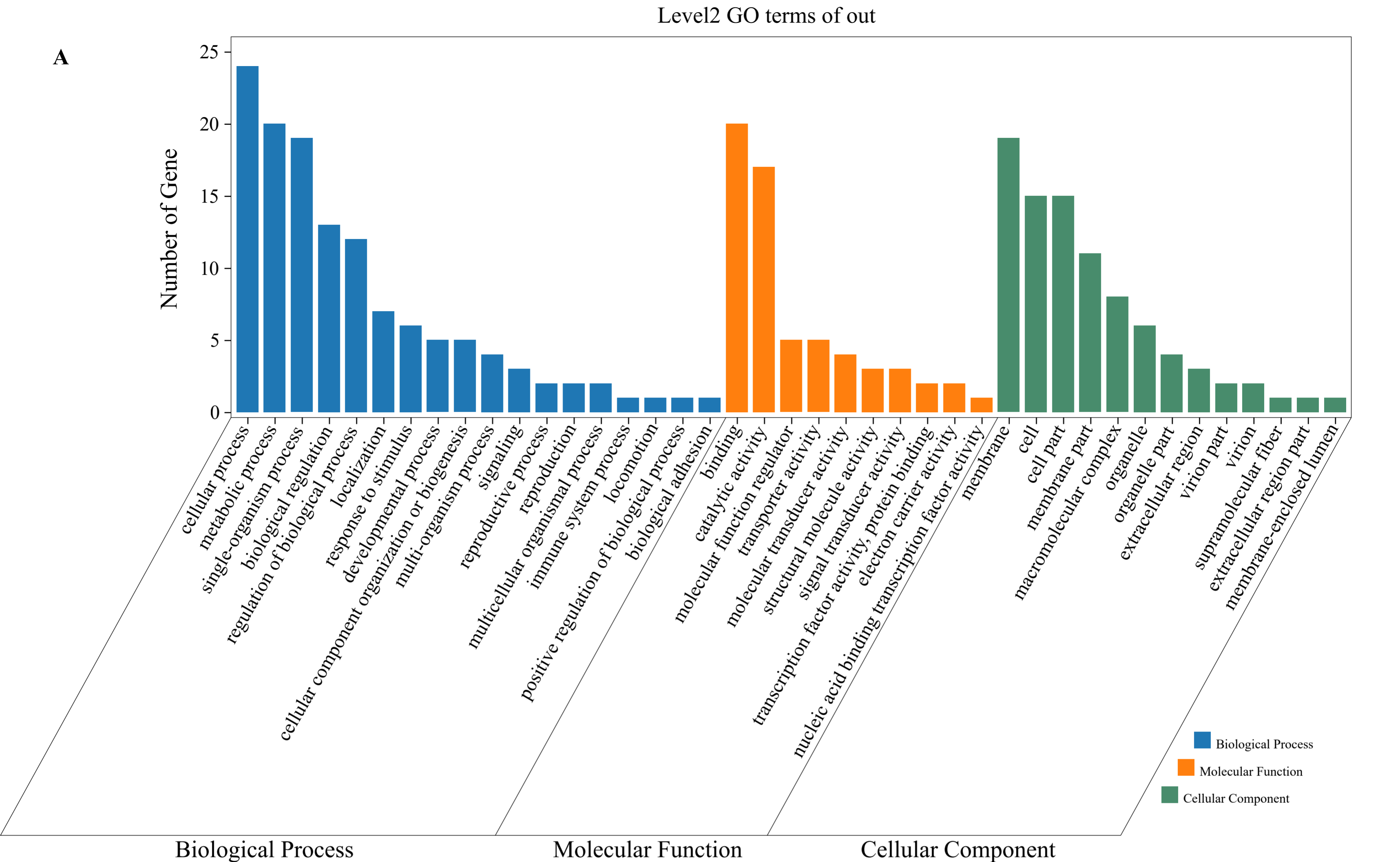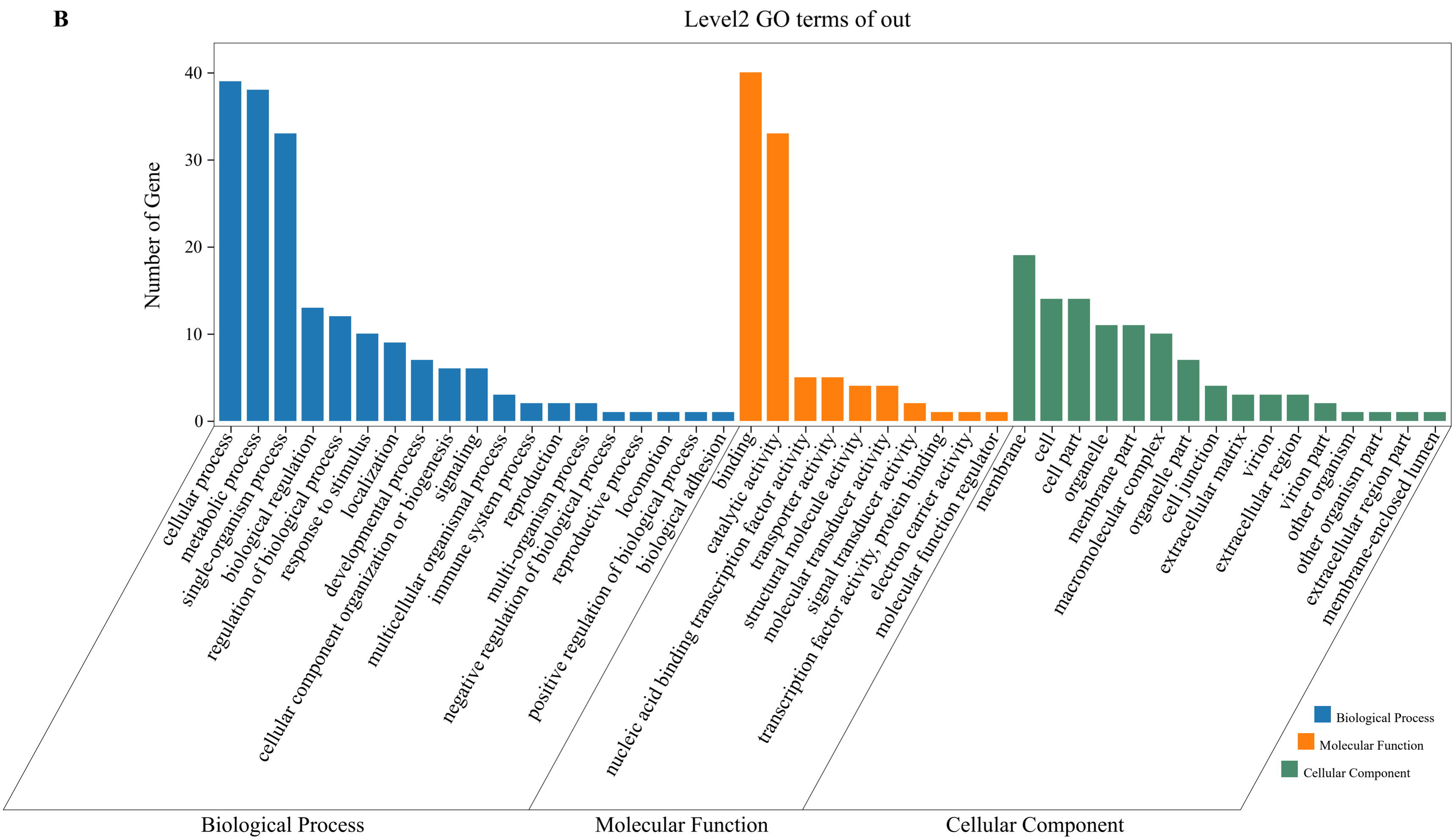

Supplement: Supplementary file 1 [file ijms-23-02715-s001.zip › Figure S4.pdf]

A

Top 20 of KEGG Enrichment

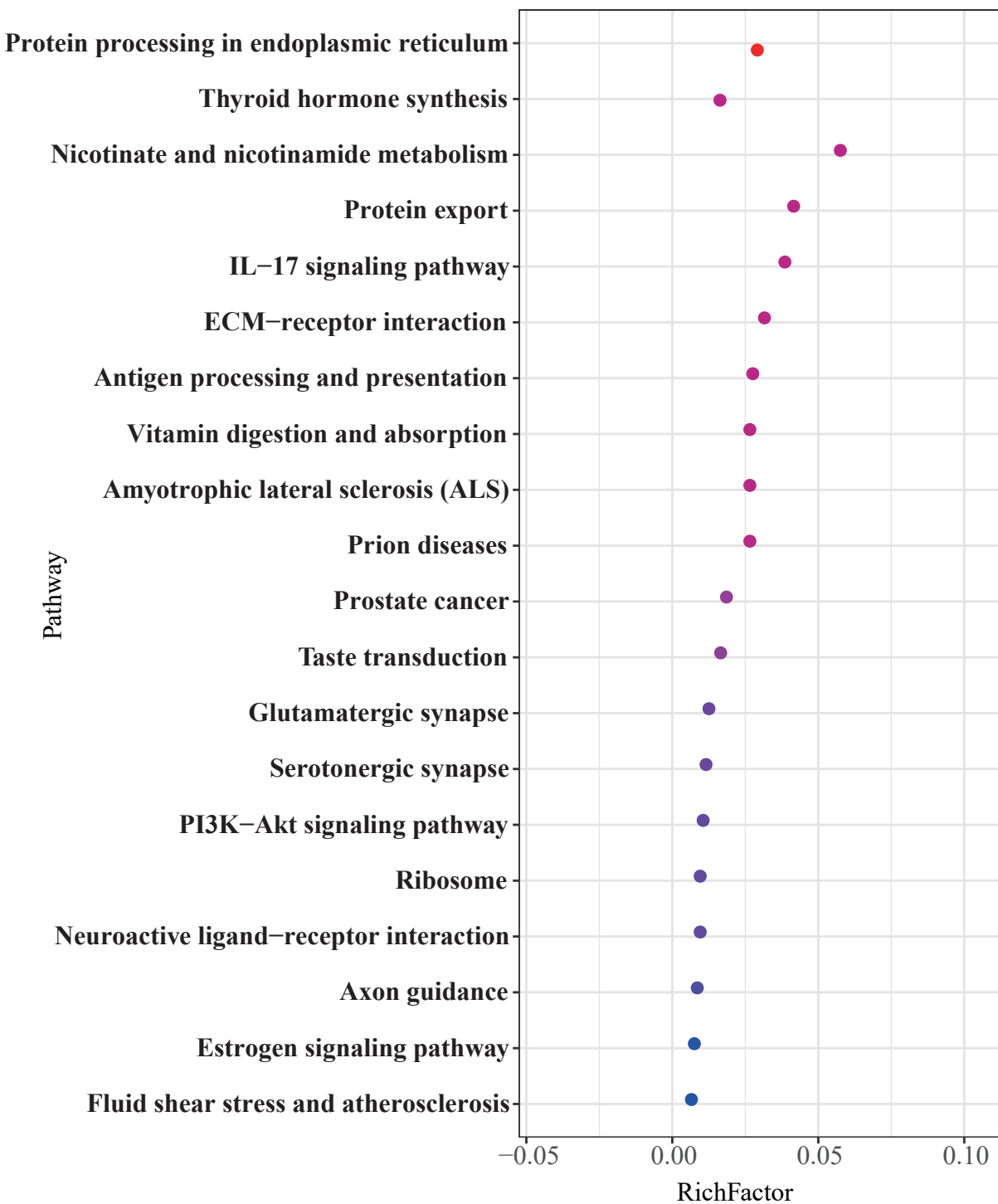

B

Top 20 of KEGG Enrichment

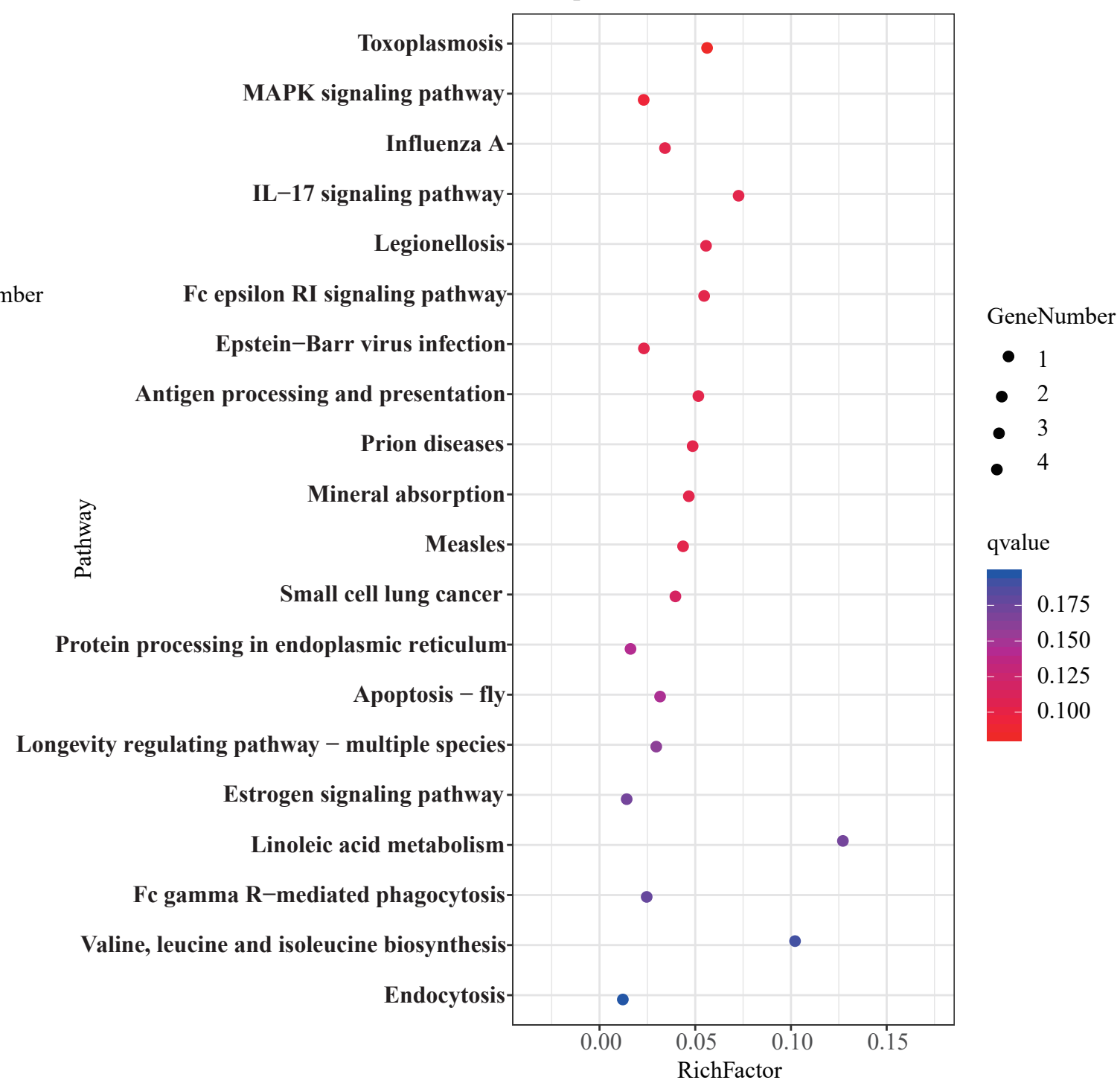

Supplement: Supplementary file 1 [file ijms-23-02715-s001.zip › Figure S5.pdf]

A

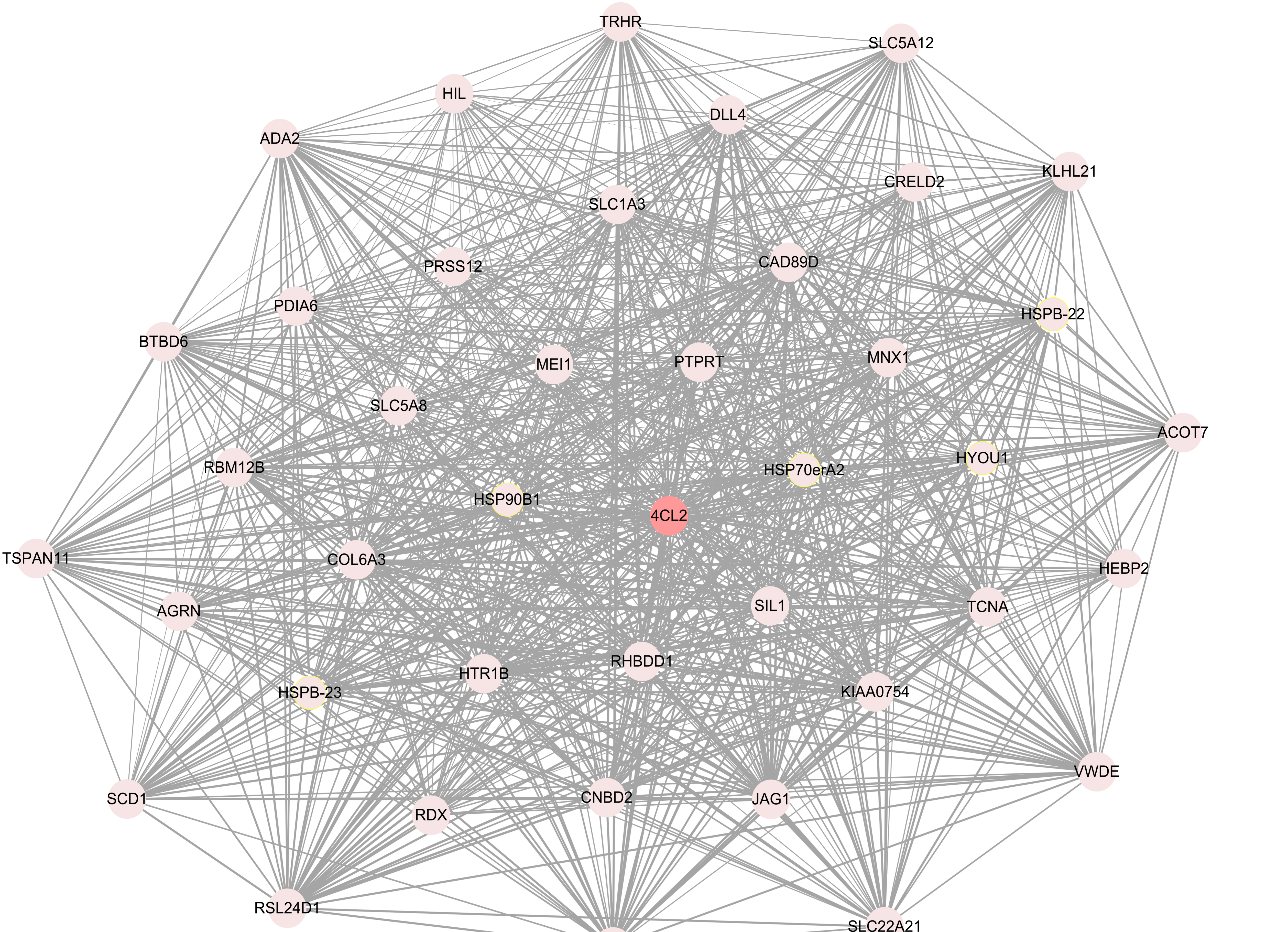

B

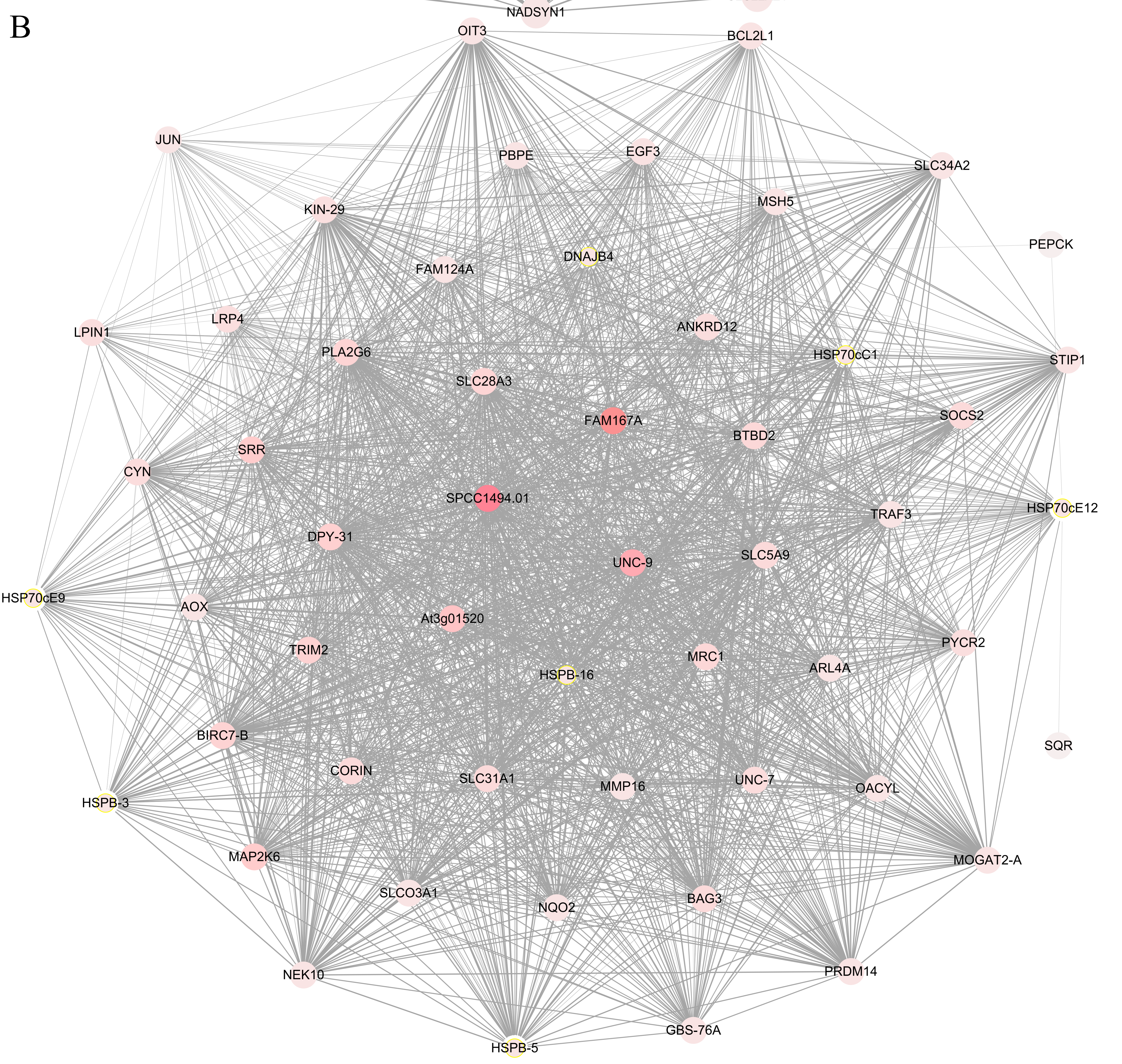

Supplement: Supplementary file 1 [file ijms-23-02715-s001.zip › Figure S6.pdf]
